# Transplant of kidneys with small renal cell carcinoma in incompatible, heavily immunosuppressed recipients

**DOI:** 10.1308/003588412X13373405384738

**Published:** 2012-09

**Authors:** AM Ali, P Rajagoppal, A Sayed, N Hakim, T David, P Papalois

**Affiliations:** Imperial College Healthcare Trust,UK

**Keywords:** Small renal cell carcinoma, Incompatible kidney transplant, Live donor

## Abstract

Renal cell carcinoma (RCC) is considered a contraindication for transplant. However, an increasing number of cases of transplant kidneys with RCC have been reported with encouraging results. We present our experience of two cases of transplanting kidneys with small RCCs. Donors and recipients were aware of the presence and possible consequences of RCC in the transplanted kidney before transplantation. Cases were discussed in the multidisciplinary team meetings. Regular, 6–12 monthly follow-up of donors and recipients was carried out with ultrasonography and/or computed tomography to detect recurrence of RCC or new tumours in the recipients’ transplant kidneys or the donors’ native kidneys. The outcome was recorded. There were no suspicious masses in the any of the kidneys during the follow-up period. The transplant kidneys are functioning.

Due to the shortage of organ supply, dependence on live kidney donation is reducing and restrictions for accepting donors are becoming less stringent. Donor safety is nevertheless of paramount importance. Donors therefore undergo extensive evaluation prior to surgery. This includes computed tomography (CT) renography or magnetic resonance angiography (MRA). These investigations lead to increased diagnosis of unexpected pathologies in donors.^1^

One of these diagnoses is the presence of small (<4cm) renal cell masses and renal cell carcinomas (RCCs). This is an overview of the management of RCC discovered either during the evaluation process of the kidney donors or after donor nephrectomy in one of the UK major centres of kidney transplantation.

## Methods

A retrospective review of the last five years of living donor kidney transplants was carried out. Two cases with RCC in the transplanted kidneys were identified.

### Case 1

In 2006 an ABO incompatible transplant took place between a 45-year-old healthy female kidney donor and her 57-year-old husband with end stage renal disease (ESRD) secondary to Alport syndrome. The donor also had a history of hypertension and post-immunosuppression lymphoproliferative disorder. The recipient had desensitisation with rituximab and plasma exchange according to the local ABO incompatible transplant protocol. During the donor nephrectomy, a 0.5cm cystic lesion was discovered in the lower pole of the kidney. The lesion was excised with part of the surrounding healthy looking tissue and a frozen section procedure was performed, which proved the lesion to be clear cell RCC with a cancer free surgical margin.

The case was discussed with the recipient, and the risk of recurrence of the RCC in the transplant kidney and consequences of this were explained thoroughly. The recipient decided to go ahead with the transplant, accepting the risks explained to him.

### Case 2

In 2008 a 72-year-old healthy female potential donor was discovered to have a 14mm suspicious exophytic mass at the posterior medial aspect of the upper pole of the left kidney during the donor workup with pre-operative magnetic resonance angiography. CT of the chest, abdomen and pelvis confirmed the presence of the lesion and excluded the presence of any other suspicious lesions.

The potential recipient was the donor’s 71-year-old sister with ESRD secondary to focal segmental glomerulosclerosis with a history of a right mastectomy for a breast carcinoma. This was performed five years prior to the transplant and the patient had been free of breast cancer during that period. However, the patient had a B cell positive cross-match, and desensitisation with rituximab and plasma exchange was necessary before the transplant, according to the local protocol.

The case was discussed with the donor and the recipient, explaining the possible diagnosis of RCC in the suspicious mass as well as the possible recurrence of the RCC in the transplant kidney and its consequences. Both the donor and recipient accepted the risk and the transplant was carried out.

In these two cases, regular follow-up by ultrasonography every 3 months and CT every 6–12 months was carried out for both the donor and recipient for recurrence of the original tumour or development of a new tumour. The outcomes of the donors and recipients were recorded.

## Results

Both donors are alive with no clinical or radiological evidence of local, contralateral or distant recurrence of the tumour. Neither of the recipients have developed any suspicious lesions in the transplant kidneys or distant metastasis elsewhere. However, the recipient in the first case developed a haemangiosarcoma in a right brachiocephalic arteriovenous fistula 18 months after the transplant. This required a high above-elbow amputation. Both grafts have been functioning well.

## Discussion

Several studies have demonstrated a significant survival advantage with transplantation over dialysis, particularly in elderly patients and in those with co-morbidities.^2^

There are few reports of transplanting kidneys with small RCC. Brook *et al* reported 43 transplant patients with previously diagnosed small RCC.^3^ These patients had significant co-morbidities. Only one recurrence of RCC in the transplant kidney after nine years of follow-up was reported. Furthermore, Sener *et al* reported no recurrence of the RCC in 3 patients after a median follow-up of 15 months.^4^

In our study as well as in other studies, the kidney recipients were high risk dialysis patients in whom the morbidity of dialysis outweighed the risks of transplanting kidneys containing a small RCC. Transplantation provides a substantial survival advantage compared with dialysis in this group of patients.^5^

Our approach in the above cases was to engage in thorough multidisciplinary team discussion, to explain to both the donors and recipients regarding all the possible consequences and to follow up the donors and recipients closely with ultrasonography, CT and biopsy of any suspicious lesions. We recommend that these kidneys are used only in high risk recipients where the risks of dialysis outweigh the risks of tumour recurrence. There is no need for a change in immunosuppression protocol in these cases.

## Conclusions

The outcome after kidney transplants following the removal of a small RCC is comparable with that of a normal kidney. However, this should be restricted to high risk recipients in whom the risks of dialysis outweigh the risks of tumour recurrence. Strict follow-up is essential.
